# A High Throughput Assay for Screening Host Restriction Factors and Antivirals Targeting Influenza A Virus

**DOI:** 10.3389/fmicb.2016.00858

**Published:** 2016-06-03

**Authors:** Lingyan Wang, Wenjun Li, Shitao Li

**Affiliations:** ^1^Department of Physiological Sciences, Oklahoma State UniversityStillwater, OK, USA; ^2^Department of Prosthodontics, School of Stomatology, Peking UniversityBeijing, China

**Keywords:** flu, antiviral, high throughput, dot blot, screening

## Abstract

Influenza A virus (IAV) is a human respiratory pathogen that causes seasonal epidemics and occasional global pandemics with devastating levels of morbidity and mortality. Currently approved treatments against influenza are losing effectiveness, as new viral strains are often refractory to conventional treatments. Thus, there is an urgent need to find new therapeutic targets with which to develop novel antiviral drugs. The common strategy to discover new drug targets and antivirals is high throughput screening. However, most current screenings for IAV rely on the engineered virus carrying a reporter, which prevents the application to newly emerging wild type flu viruses, such as 2009 pandemic H1N1 flu. Here we developed a simple and sensitive screening assay for wild type IAV by quantitatively analyzing viral protein levels using a Dot Blot Assay in combination with the LI-COR Imaging System (DBALIS). We first validated DBALIS in overexpression and RNAi assays, which are suitable methods for screening host factors regulating viral infection. More importantly, we also validated and initiated drug screening using DBALIS. A pilot compound screening identified a small molecule that inhibited IAV infection. Taken together, our method represents a reliable and convenient high throughput assay for screening novel host factors and antiviral compounds.

## Introduction

Influenza A virus is a highly transmissible respiratory pathogen and presents a continued threat to global health with considerable economic and social impact (Kawaoka, 2006; Hale et al., 2010; Fukuyama and Kawaoka, 2011; Tscherne and Garcia-Sastre, 2011). In spite of vaccines and antiviral drugs flu remains a major burden to human health in the US and worldwide. In times of flu pandemics these numbers can significantly rise and the threat to the health care system itself is a serious concern (Schultz-Cherry and Jones, 2010; Medina and Garcia-Sastre, 2011; Govorkova and McCullers, 2013). There are two types of drugs used to treat flu infection. The amantidines block entry of virions through the acidified endosomal compartment. The other anti-flu treatments are sialic acid mimics, which inhibit the activity of viral neuraminidase and prevent release of new viral particles. Both types of anti-influenza therapies directly target the activity of viral genes. However, there has been a rapid worldwide increase in resistance to these drugs among circulating flu viruses (Medina and Garcia-Sastre, 2011; Govorkova and McCullers, 2013; Kamali and Holodniy, 2013; Samson et al., 2013). Thus, it is pressing to develop new antiviral strategies.

IAV is a member of the *Orthomyxoviridae* family and possesses eight segments of negative-sense single-stranded RNA genome that encode 11 major viral proteins. IAV initiates infection by hemagglutinin (HA) binding sialic acid sugars on plasma membrane. Then viral particle are ferried into the late endosome by endocytosis. The acidic environment in the late endosome induces a conformational change of HA to mediate membrane fusion. The matrix 2 ion channel protein acidifies the core of the virus, which causes to the release of viral ribonucleoprotein complexes (vRNPs) from matrix 1 proteins into cytoplasm. vRNPs consist of nucleoprotein (NP) and heterotrimeric viral polymerase subunit proteins [polymerase acid (PA), polymerase basic 1 and 2 (PB1 and PB2)]. The vRNPs are transported into nucleus, where the RNA-dependent RNA polymerase begins replication and transcription. Viral RNA is exported into the cytoplasm and translated. After new viral particles are assembled, the mature virus buds off from the cell once its neuraminidase has cleaved sialic acid residues from the host cell. Briefly, the influenza virus life cycle can be divided into three distinct stages (entry, replication, and egress), each of which could be targeted by small molecule inhibitors.

Recently, three types of high throughput reporter assays have been developed for indirect measurement of IAV infection (Beyleveld et al., 2013). The first type of assay is based on IAV minigenome system. Cells are transfected with a plasmid that will generate a virus-like RNA, which encodes a reporter gene under control of the viral polymerase regulatory sequences (Su et al., 2010; Ozawa et al., 2013; Wang et al., 2015). The second way is to generate an IAV virion in which one viral gene is replaced by a reporter gene. The engineered virus is replication-incompetent. For example, König et al. generated a recombinant influenza virus where the open reading frame (ORF) of HA was replaced with *Renilla* luciferase (König et al., 2010). The virus needs to grow in a cell-line that stably expresses HA to complement the loss of the viral segment encoding HA. The third type is to generate a replication competent reporter IAV (Manicassamy et al., 2010; Heaton et al., 2013; Pan et al., 2013; Karlsson et al., 2015). One of examples is the PR8 IAV carrying a *Gaussia* luciferase (GLuc) on its PB2 segment. The GLuc ORF was inserted after the PB2 ORF, which contained a mutated packaging signal on its 3′ end. A functional PB2 packaging signal was then repeated on the 3′ end of the GLuc ORF (Heaton et al., 2013). However, all these assays require expression of a reporter gene as an indicator of viral polymerase activity. The feasibility and variability of this requirement limits its application to wild type IAVs, such as emerging new viral strains. Thus, there is a need for developing a general non-reporter based screening assay.

Here, we report a novel assay suitable for high throughput screening inhibitors targeting IAV infection. Our assay directly measures wild type virus infection activity by quantitatively analyzing viral protein levels using DBALIS. We validated the assay in overexpression and RNAi experiments. Furthermore, we initiated a pilot compound screening and identified a small molecule that inhibits IAV infection. Hence, our data demonstrate that DBALIS is suitable for high throughput screening of novel host restriction factors and anti-influenza drugs.

## Materials and methods

### Cells and viruses

HEK293 cells (ATCC, #CRL-1573) were maintained in Dulbecco's minimal essential medium (Life Technologies, #11995-065) containing antibiotics (Life Technologies, #15140-122) and 10% Fetal Bovine Serum (Life Technologies, #26140-079). A549 cells (ATCC, #CCL-185) were cultured in RPMI Medium 1640 (Life Technologies, #11875-093) plus 10% fetal bovine serum and 1X MEM Non-Essential Amino Acids Solution (Life Technologies, # 11140-050).

Influenza A/Puerto Rico/8/34 (PR8) was purchased from Charles River Laboratories (#10100374). Influenza A/WSN/33 (WSN/33) and PR8 IAV carrying a Gluc gene (PR8-Gluc; Heaton et al., 2013) were generous gifts from Dr. Peter Palese (Mount Sinai School of Medicine, NY, NY). IAVs was propagated in specific pathogen-free fertilized eggs Premium Plus (Charles River Laboratories, Wilmington, MA) according to previously published protocols (Szretter et al., 2006).

### Plaque assay

IAV was tittered by plaque assay as described by Matrosovich et al. (2006). Briefly, 1.2 × 10^6^ MDCK cells/ml were split into six-well-plates. After 2X washes with DMEM, serial dilutions of flu virus were adsorbed onto the cells for 1 h. Cells were covered with DMEM containing 2 × Avicel RC591 NF (FMC Biopolymer, Philadelphia, PA) and 1 μg/ml TPCK-trypsin (Thermo Fisher Scientific, #20233). Crystal violet staining was performed 48 h.p.i., and visible plaques were counted.

### Dot blot assay using LI-COR imaging system

**Figure 1:**
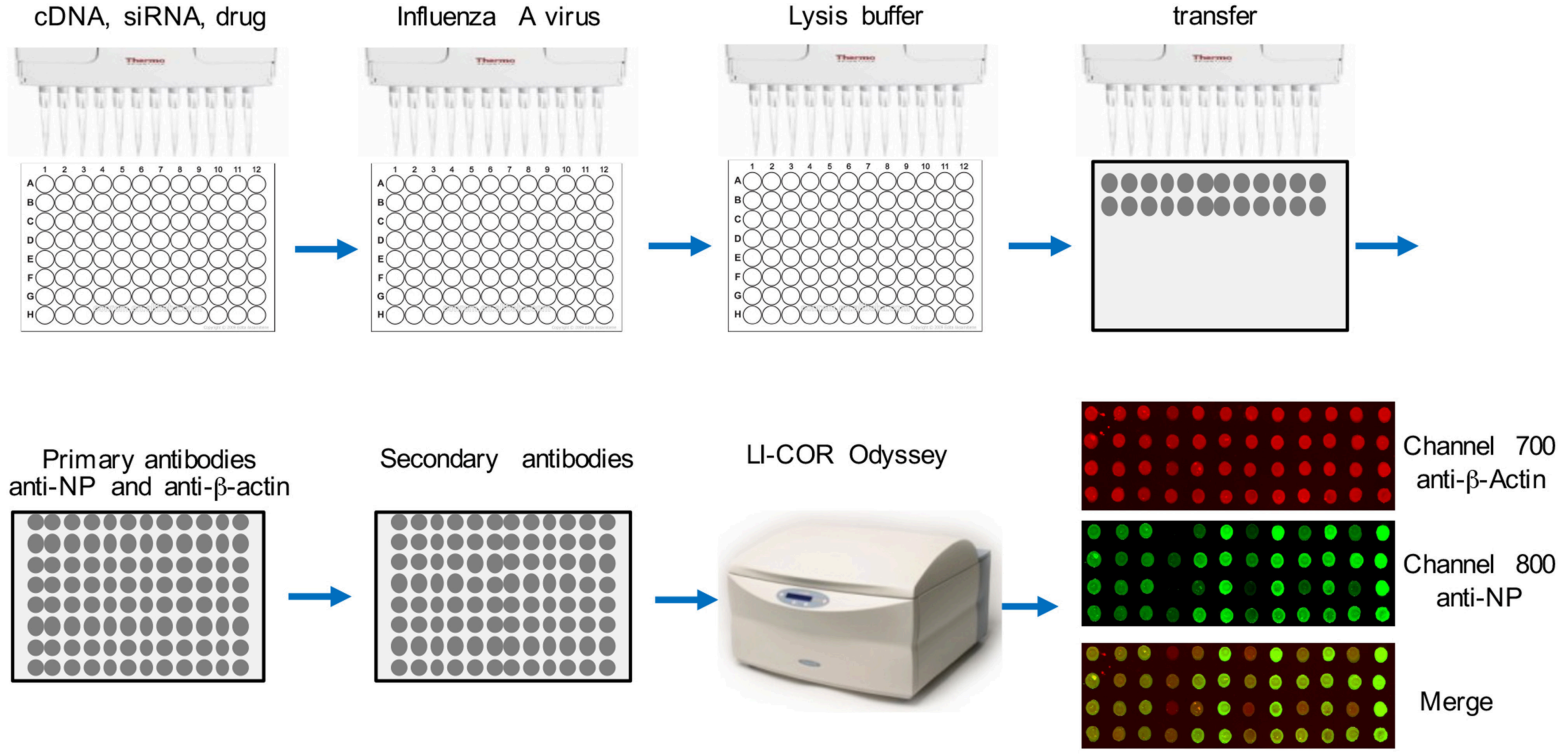
**Flowchart of DBALIS**. The procedures are detailed in the Materials and Methods.

The flowchart of this assay is depicted in Figure 1. Cells were seeded into a 96-well-plate. After transfection or treatment, cells were infected with influenza virus. After 16 h, about 2 × 10^4^ cells in the well were lysed in 25 μl TAP lysis buffer [50 mM Tris-HCl (pH 7.5), 10 mM MgCl_2_, 100 mM NaCl, 0.5% Nonidet P40, 10% glycerol, Complete EDTA-free protease inhibitor cocktail tablets (Roche, #11873580001)] for 30 min at 4°C. Then the plate was centrifuged for 30 min at 2000 g. Two-microliter lysate from each well was transferred onto a nitrocellulose membrane using a multichannel pipettor. After air-drying for 30 min at room temperature, the dot blot membrane was blocked with Odyssey Blocking Buffer (LI-COR Biosciences, # 927-40000) for 1 h. Then the blot was washed with 1X TBS buffer for 30 min and incubated with anti-β-actin (Abcam, #ab8227, 1:1000 dilution) and anti-NP (BEI resources, # NR-4282, 1:1000 dilution) at 4°C for 16 h. Then the blot was 3X washed with 1X TBS (each time for 10 min) and incubated with secondary antibodies, goat anti-rabbit IRDye@680 (1:10000 dilution) and goat anti-mouse IRDye@800 (1:10000 dilution) at room temperature for 1 h. After washing 3X with 1X TBS (each time for 10 min), the dot blot membrane was placed on the reading glass platform of the LI-COR Odyssey® Infrared Imaging System (LI-COR Biotechnology, NE) with the reader is set for a 96-well-plate reading. The blot was scanned using the 800 nm channel for viral NP detection and the 700 nm wavelength for anti-β-actin, which is the control for cell numbers. Blank negative control dots were set using the LI-COR Odyssey software. Each dot was quantified using LI-COR Odyssey software, ImageStudio^TM^ according to the manufacturer's instructions.

### Real-time PCR

Total RNA was prepared using RNeasy columns (Qiagen, # 74136). One microgram RNA was transcribed into cDNA using QuantiTect reverse transcription kit (Qiagen, # 205311). Twenty microliter SYBR Green PCR reaction mix (Roche Applied Science), containing 1/10 of the synthesized cDNA plus an appropriate oligonucleotide primer pair, were run on the LightCycler 480 (Roche). The comparative Ct method was used to determine relative mRNA expression of genes as normalized by the housekeeping gene, GAPDH. The primer sequences are: TRIM32: sense, 5′-CCGGGAAGTGCT AGAATGCC-3′, antisense, 5′-CAGCGGACACCA TTGATGCT-3′; FKBP8: sense, 5′-GGAGTACAG TGAGGCCATCC-3′, antisense, 5′-GGATCG TCTTGTTGGAAGGT-3′; GAPDH, sense, 5′-AGG TGAAGGTCGGAGTCA-3′, antisense, 5′-GGTCAT TGATGGCAACAA-3′.

### Plasmid transfection and RNA interference (RNAi)

Control vector or eight plasmids were transfected individually into HEK293 cells using Lipofectamine 2000 Transfection Reagent (Life Technologies, # 11668-019) according to the manufacturer's protocol. After 24 h, cells were infected with IAV. These genes were used for this study: TRIM32 (BC003154), STUB1 (BC0075456), IQSEC (BC010267), GALK1 (BC 001166), PDCD6 (BC050597), HAUS6 (NM 001270890.1), FKBP8 (NM_012181.3), and HAX1 (BC005240.1).

The control RNAi used was Dharmacon siGENOME Non-Targeting Control siRNA #2 (# Dharmacon D-001210-02). A549 cells were transfected with siRNA duplexes using Lipofectamine RNAiMAX Transfection Reagent (Life Technologies, # 13778030) according to manufacturer's protocol. Knockdown was allowed to proceed for 72 h before cells were infected with IAV. RNAi target sequences used: TRIM32: sense, 5′-GACCGTGGTAACTATCGTATA-3′; FKBP8: sense, 5′-GGAAGAGGATGACCTGAGTGA-3′.

### Reporter assay

Cells were aliquoted in 96-well-plates for 24 h prior to IAV infection. Cells were infected with PR8-GLuc. After 1 h the viral inoculum was aspirated, cells were washed twice and incubated at 37°C with 0.2% BSA-DMEM without serum. Cells were lysed 16 h post of infection and the luciferase assay was performed using BioLux Gaussia Luciferase Assay Kit (NEB, Ipswich, MA).

### Cell viability

Cell viability was assessed by using the CellTiter-Glo Luminescent Cell Viability Assay (Promega) according to the manufacturer's instructions. Briefly, 2 μl lysate was added into 20 μl CellTiter-Glo reagent preloaded in a 96-well-plates. Then the plate was subjected to luminescence measurement.

### Statistical analysis

*Z*′ factor is a dimensionless calculation used to assess the quality of a population of sample compounds tested (Zhang et al., 1999). The *Z*′ value was calculated as follows: *Z*′ = 1 – 3 × (*SD* of positive control + *SD* of negative control)/|mean of positive control − mean of negative control|, where *SD* represents the standard deviation. *Z*′ value between 0.5 and 1.0 is considered as an excellent assay for high throughput screening.

## Results

### Establishment of DBALIS

**Figure 2:**
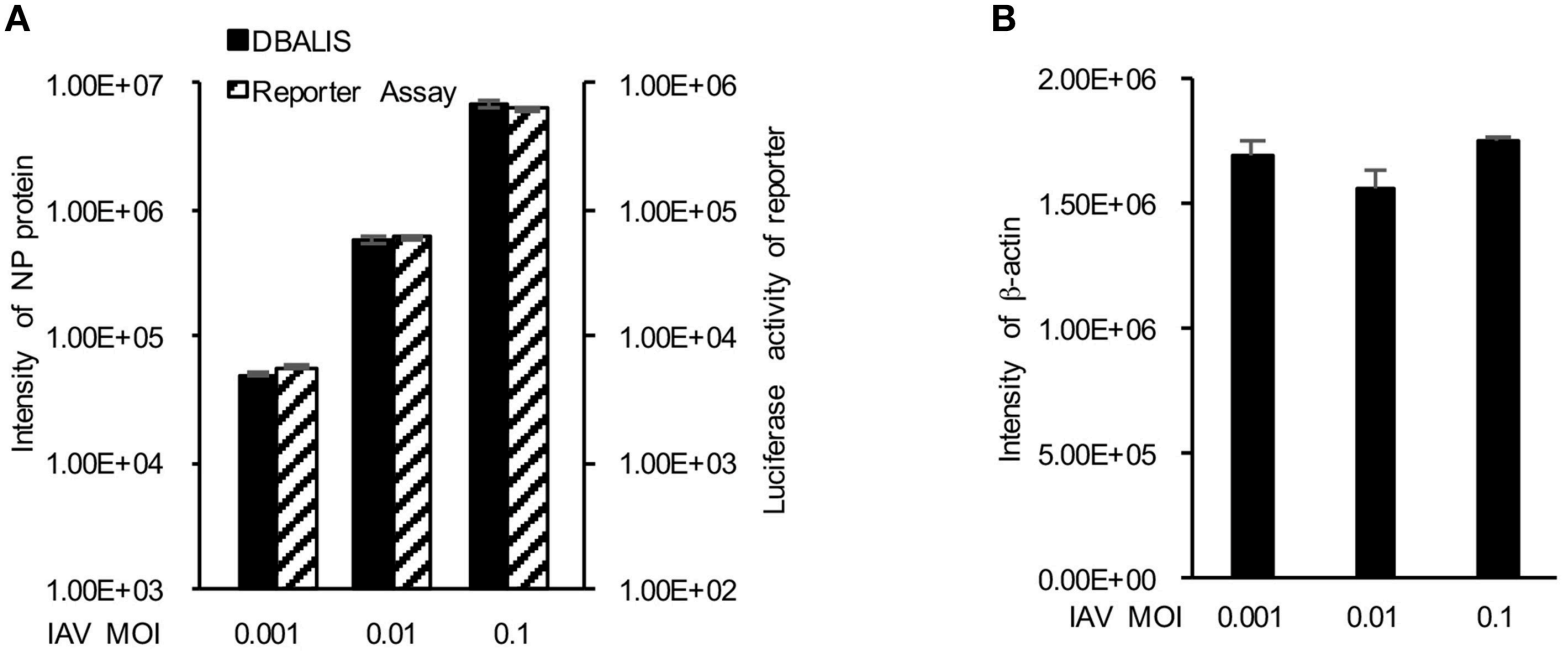
**Comparison of DBALIS with viral reporter assay. (A)** Lung epithelial A549 cells in 96-well-plates were infected with 0.001, 0.01, or 0.1 MOI of IAV PR8-Gluc for 16 h. Two microliter of the same cell lysates were used for DBALIS and reporter assays, respectively. **(B)** DBALIS determined the Intensity of β-actin in the same samples used in **(A)** All experiments were repeated three times and error bars are indicated.

We first examined the feasibility of DBALIS on IAV infection. To compare DBALIS and reporter-based assay in parallel, we used influenza PR8-GLuc virus. Lung epithelial A549 cells in 96-well-plates were infected with 0.001, 0.01, or 0.1 MOI of IAV PR8-Gluc for 16 h. Two microliter of cell lysates were applied onto nitrocellulose membrane for the Dot Blot. Viral NP and host β-actin protein levels have been used to indicate viral infection activity and cell number, respectively. The dot membrane was incubated with anti-NP and anti-β-actin antibodies for 16 h in a shaker at 4°C. The intensities of NP in each dot were analyzed by ImageStudio^TM^. Using the same cell lysates, luciferase activity was also determined by reporter assay. As shown in Figure 2A, both assays demonstrate that the reporter activity and the protein levels of NP were increased roughly by a factor of 10, which is highly correlated to the titers of IAV. The protein levels of β-actin had minimal changes (Figure 2B), indicating comparable cell numbers in these samples. Therefore, DBALIS exhibited similar sensitivity, robustness, and correlation as the reporter assay that had previously been established for high throughput screens. Taken together, we present a novel assay for quantitative measurement of IAV infection.

### Functional validation of DBALIS in overexpression and RNA interference assays

**Figure 3:**
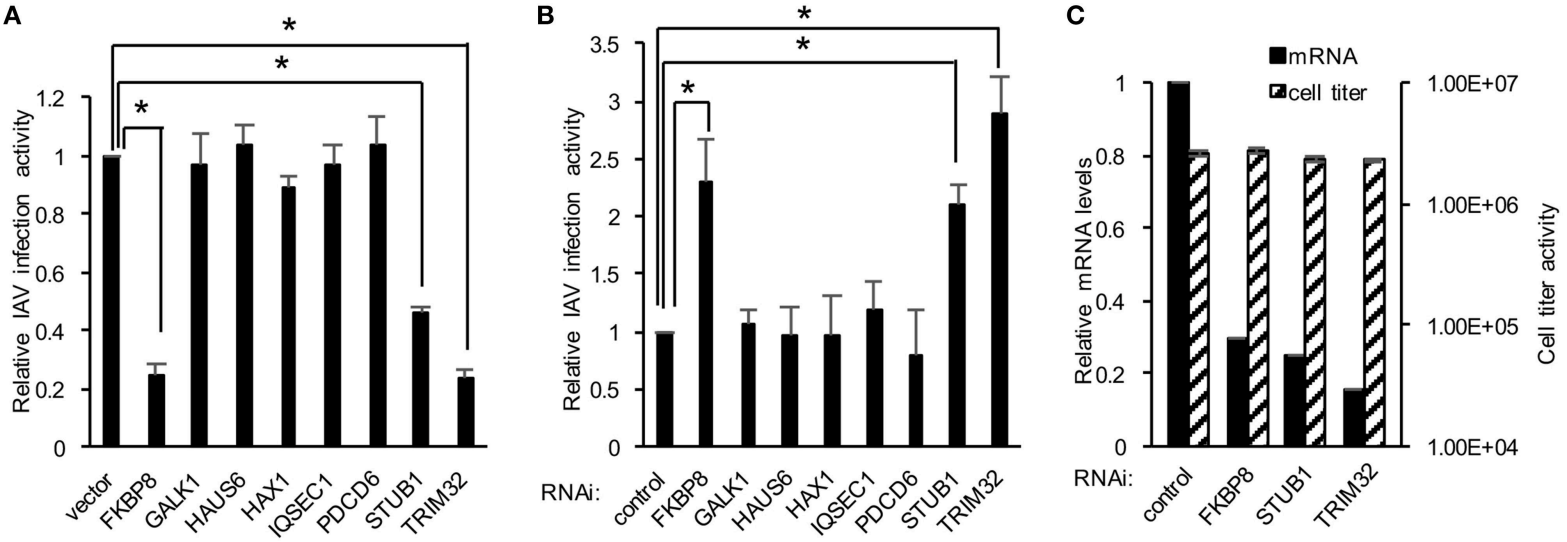
**Applying DBALIS to gain- and loss- of function screenings. (A)** HEK293 cells were transfected with control vector or the indicated genes for 24 h. Cells were infected with 0.1 MOI IAV PR8 for 16 h. The ratio of NP to β-actin was calculated. The relative viral infection activity was determined by comparing the NP/actin ratio to the control vector that was set as one. All experiments were repeated three times and two-tailed student *t*-test was performed. An asterisk indicates *P* < 0.05. **(B)** A549 cells were transfected with scrambled control siRNA and indicated siRNA oligos. After 72 h, cells were infected with 0.1 MOI IAV PR8 for 16 h. The relative IAV infection activities were determined by DBALIS. All experiments were repeated three times and two-tailed student *t*-test was performed. An asterisk indicates *P* < 0.05. **(C)** Knockdown efficiency and cell viability were determined by real-time PCR and CellTiter-Glo®, respectively. A549 cells were transfected with scrambled control siRNA or the indicated siRNA oligos. After 72 h, mRNA levels and cell viability were examined. All experiments were repeated three times.

We next explored the application of DBALIS on gain- and loss- of function screenings of host factors targeting IAV. We first selected eight host factors that have been shown to interact with influenza PB1 protein previously in our lab (Fu et al., 2015). We overexpressed each candidate gene or a control vector in HEK293 cells. After 24 h cells were infected with 0.1 MOI of PR8 IAV for 16 h. The ratio of NP to β-actin was calculated. The relative viral infection activity was determined by comparing to the NP/actin ratio of control vector. The NP/actin ratio of vector was set at one. DBALIS found that overexpression of TRIM32, STUB1 and FKBP8 reduced influenza infection activity from two to four fold (Figure 3A), indicating their role in viral restriction. We also performed RNA interference (RNAi) targeting the above eight host factors. Scrambled control siRNA or siRNA duplex of each gene was transfected into A549 cells for 72 h. Then cells were infected with 0.1 MOI of PR8 IAV for 16 h. DBALIS showed that knockdown of TRIM32 increased NP protein level by a 2.9-fold over control RNAi (Figure 3B). RNAi depletion of STUB1 and FKBP8 also increased viral NP level by 2.1 and 2.3-fold, respectively. Knockdown efficiency of these three genes was about 70 to 85% (Figure 3C). CellTiter Glo® assays showed that RNAi had marginal effects on cell viability (Figure 3C). TRIM32 and STUB1 are genes known to limit influenza infection (Fu et al., 2015). Thus, the confirmation of known anti-influenza genes validates the reliability of DBALIS. Taken together, these data prove the concept of DBALIS in the successful application of the technique to high throughput screening of host factors regulating IAV infection.

### Functional validation of DBALIS in an anti-influenza drug assay

**Figure 4:**
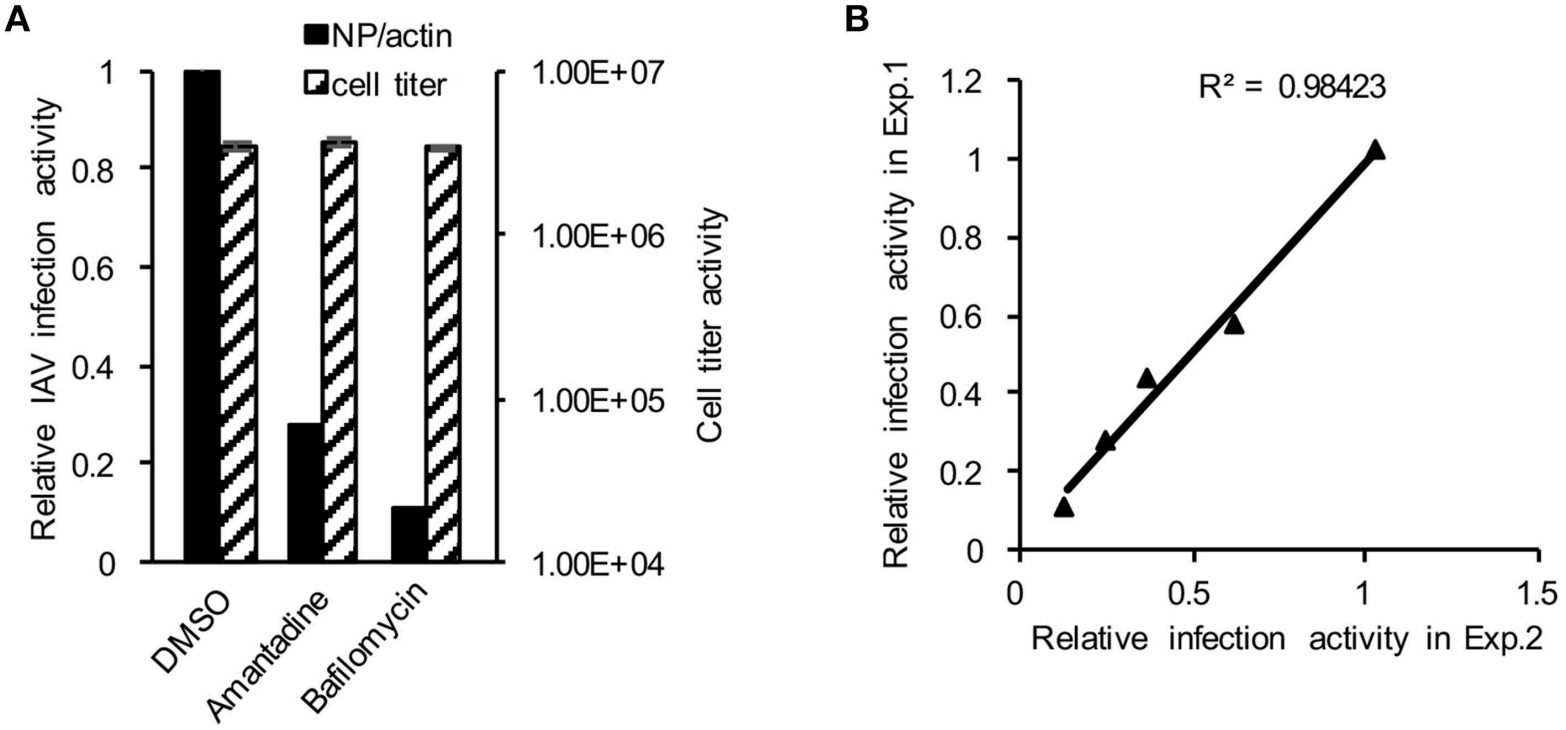
**Validation of DBALIS in drug screenings. (A)** A549 cells were incubated with 50 μM amantadine hydrochloride and 25 nM bafilomycin A1 for 4 h, then infected with PR8 IAV for 16 h. Viral infection activity and cell viability were determined by DBALIS and CellTiter Glo®, repectively. **(B)** Determination of *R*^2^-value. A549 cells were treated with various concentration of Amantadine for 72 h. IAV infection activity was then determined by DBALIS. *R*^2^ was calculated in two independent experiments (each experiment was in triplicates). The triangle stands for the data point of IAV reporter activity.

To establish the application of DBALIS for screening inhibitors targeting IAV infection, we used DBALIS to examine the effects of two known anti-influenza drugs, amantadine and bafilomycin A1. Amantadine is a known inhibitor of IAV M2 ion channel (Wang et al., 1993). Bafilomycin A1 is an endosomal acidification inhibitor that has been shown to block viral endocytic entry (Ochiai et al., 1995). A549 cells were incubated with 50 μM amantadine hydrochloride or 25 nM bafilomycin A1 for 4 h, then infected with PR8 IAV. DBALIS showed both drugs inhibited IAV infection with minimal effects on cell viability (Figure 4A). To determine the robustness of the assay, we examined the correlation of reproducibility of the assay using serial dilutions of bafilomycin A1. The high *R*^2^-value (*r*^2^ = 0.98, *P* < 0.003) indicates the high reproducibility of the assay (Figure 4B), validating the DBALIS in drug screening.

### Pilot screening identifies UCN-01 as a novel influenza inhibitor

**Figure 5:**
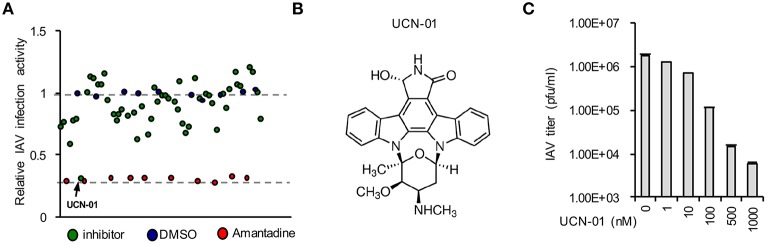
**Drug screening identified UCN-01 as a novel inhibitor of influenza A virus. (A)** A549 cells were treated with DMSO, Amantadine, or 50 small molecules for 4 h Then cells were infected WSN/33 IAV for 16 h. Relative infection activity was determined by DBALIS. Blue circles represent negative controls of DMSO while red circles are the positive controls of Amantadine. The arrow indicates UCN-01. **(B)** Chemical structure of UCN-01. **(C)** A549 cells pretreated with designated concentrations of UCN-01 were infected with 0.001 MOI of WSN/33 IAV. Culture supernatants containing IAV were titered on MDCK cells and plaques were enumerated.

We initiated a pilot screen of 50 small molecules targeting various signaling pathways. A549 cells were seeded into a 96-well-plate. In the plate, 10 wells were treated with negative and positive controls, DMSO, and Amantadine, respectively. After treatment with inhibitors for 4 h, cells were infected influenza A/WSN/33 (WSN/33). After screening using DBALIS (Figure 5A), we utilized the *Z*′ factor to evaluate the quality of the method in high throughput assays. The Z′ factor is reported to reflect both the assay signal dynamic range and the data variation associated with the signal measurements (Zhang et al., 1999). The mean and standard deviation of positive and negative controls were 0.25, 0.032, 1.051, 0.036, respectively. The Z′ factor of our drug screening was 0.745, a score that is considered robust and reliable, suggesting that DBALIS is suitable for high throughput screening for small molecules restricting influenza infection.

A small molecule, UCN-01 exhibited a significant inhibitory effect on NP expression while none of the compounds in the screening exhibited cytotoxicity in A549 cells (Figure 5A). UCN-01 is 7-hydroxystaurosporine, which inhibits the enzymatic activity of protein kinase C (PKC; Figure 5B) (Takahashi et al., 1987). Primary screening suggests that UCN-01 possesses anti-influenza virus activity. Therefore, we further assessed the inhibitory effect of UCN-01 by plaque assays of WSN/33 IAV. Plaque assays showed that 1 μM UCN-01 inhibited viral growth by more than 100-fold (Figure 5C). These results substantiate that UCN-01 is a bona fide inhibitor of IAV.

## Discussion

To date, most published works use reporter systems to screen host factors and inhibitors. However, the reporter system reflects viral infection indirectly and luciferase activity could be affected by several factors, such as buffer and temperature (Kricka and Deluca, 1982; Harrison et al., 2006). Moreover, the availability of existing reporter systems or the feasibility of constructing new ones interferes with the application to wild type and newly emerging IAVs. Here we develop a new assay combining Dot Blot and the LI-COR imaging system that is suitable for sensitive and quantitative high throughput screening. DBALIS is an easy and simple assay that only needs an antibody against a viral protein and the LI-COR Imaging System. Thus, DBALIS provides an advantage as it allows screening of wild type IAVs, which is potentially applicable for measurement of the infections with various types and strains of viruses. We demonstrate that this assay can be applied to gene function and small molecule screenings. We also successfully validate the use of DBALIS to examine viral infection with two wild type IAV strains (PR8 and WSN/33) in two human cell lines (HEK293 and A549). These data suggest the versatility and broad application of DBALIS.

Although DBALIS is an efficient high throughput screening method, there are several limitations. First, it requires high quality antibodies for Dot Blot. Secondly, proteins are not separated in Dot Blot, so low steric size proteins could be masked by high steric size proteins on the spot surface. Thirdly, in comparison with reporter virus-based screening, DBALIS is relative laborious and time consuming. Last, DBALIS uses β-actin as an input control. At higher total protein loads as required for the detection of low-abundance proteins, β-actin-specific antibodies are not reliable to distinguish differences in actin protein levels (Dittmer and Dittmer, 2006). Despite of these limitations, the DBALIS provides an alternative and complementary method for reporter-based screening.

Our pilot drug screening identified UCN-01 as an IAV inhibitor. UCN-01 was originally isolated from the culture broth of Streptomyces species as a PKC selective inhibitor (Takahashi et al., 1987). PKC has been implicated in viral entry processes. The entry of several enveloped viruses, including influenza viruses, poxviruses, and Herpesviruses, has been proposed to require PKC (Constantinescu et al., 1991). During entry, influenza virus traverses the endocytic pathway and has the ability to activate PKC upon binding to host cell surface receptors (Kunzelmann et al., 2000). Another PKC inhibitor, bisindolymaleimide I, also restricts IAV infection (Root et al., 2000), attesting to the potential of UCN-01 as an anti-influenza agent.

UCN-01 also shows antitumor activity, however, the precise mechanism is still not fully understood. Clinical studies show that UCN-01 causes arrest of cell cycle progression at G1/S phase at concentrations that reduce PKC activity (Akiyama et al., 1999; Abe et al., 2001). In addition, UCN-01 also induces apoptosis and sensitization to DNA-damaging agents (Fuse et al., 2005). Several phase I and II studies of UCN-01 either as monotherapy or in combination with cytotoxic agents have been reported (Fuse et al., 2005; Li et al., 2012; Ma et al., 2013). Whether the antitumor activity of UCN-01 affects IAV infection will need further investigation.

In summary, we established and validated a simple, convenient, and reliable high throughput-screening assay to monitor IAV infection. Using this system, we identified a promising candidate inhibitor of IAV. This novel screen assay will advance the discovery of novel antivirals and identification of new host factors controlling viral infection.

## Author contributions

SL conceived the project. LW and WL performed the experiments.

### Conflict of interest statement

The authors declare that the research was conducted in the absence of any commercial or financial relationships that could be construed as a potential conflict of interest.
